# Age-Related Comparisons of Evolution of the Inflammatory Response After Intracerebral Hemorrhage in Rats

**DOI:** 10.1007/s12975-012-0151-3

**Published:** 2012-03-16

**Authors:** Starlee Lively, Lyanne C. Schlichter

**Affiliations:** 1 Toronto Western Research Institute, University Health Network, Toronto, ON Canada; 2 Department of Physiology, University of Toronto, Toronto, ON Canada; 3 Toronto Western Hospital, MC9-417, 399 Bathurst Street, Toronto, ON Canada M5T 2S8

**Keywords:** Hemorrhagic stroke, Aging brain, Inflammation, Alternative activation, Collagenase, Striatum

## Abstract

In the hours to days after intracerebral hemorrhage (ICH), there is an inflammatory response within the brain characterized by the infiltration of peripheral neutrophils and macrophages and the activation of brain-resident microglia and astrocytes. Despite the strong correlation of aging and ICH incidence, and increasing information about cellular responses, little is known about the temporal- and age-related molecular responses of the brain after ICH. Here, we monitored a panel of 27 genes at 6 h and 1, 3, and 7 days after ICH was induced by injecting collagenase into the striatum of young adult and aged rats. Several molecules (CR3, TLR2, TLR4, IL-1β, TNFα, iNOS, IL-6) were selected to reflect the classical activation of innate immune cells (macrophages, microglia) and the potential to exacerbate inflammation and damage brain cells. Most of the others are associated with the resolution of innate inflammation, alternative pathways of macrophage/microglial activation, and the repair phase after acute injury (TGFβ, IL-1ra, IL-1r2, IL-4, IL-13, IL-4Rα, IL-13Rα1, IL-13Rα2, MRC1, ARG1, CD163, CCL22). In young animals, the up-regulation of 26 in 27 genes (not IL-4) was detected within the first week. Differences in timing or levels between young and aged animals were detected for 18 of 27 genes examined (TLR2, GFAP, IL-1β, IL-1ra, IL-1r2, iNOS, IL-6, TGFβ, MMP9, MMP12, IL-13, IL-4Rα, IL-13Rα1, IL-13Rα2, MRC1, ARG1, CD163, CCL22), with a generally less pronounced or delayed inflammatory response in the aged animals. Importantly, within this complex response to experimental ICH, the induction of pro-inflammatory, potentially harmful mediators often coincided with resolving and beneficial molecules.

**Electronic supplementary material:**

The online version of this article (doi:10.1007/s12975-012-0151-3) contains supplementary material, which is available to authorized users.

## Introduction

Spontaneous intracerebral hemorrhage (ICH) is associated with high mortality and morbidity: ~50% of patients die within 6 months, and ~80% of survivors do not regain functional independence [1, 2]. The greatest risk factor is increasing age, and consequent increases in chronic hypertension, amyloid angiopathy, and the use of anti-thrombotic drugs, followed by hypertension [3–7]. Thus, ICH incidence is expected to rise with the aging population demographic and increasing hypertension rates in younger people [8]. In young adults, hypertensive ICH results in smaller hematoma volumes and better survival rates, but more severe disabilities, suggesting age-related differences in the underlying pathology [9]. Most experimental ICH studies use young healthy animals, but age-related differences are emerging, e.g., evidence that aged animals have altered glial scar formation and microglial recruitment [10, 11], reduced lesion resolution [11], increased white matter damage [12], more severe edema and autophagy [10, 13], and poorer functional recovery [10, 13].

ICH is often described as having acute, secondary, and resolution phases. In the acute injury phase (first few hours), blood extravasation forms a hematoma, resulting in the physical destruction of adjacent tissue and mass effects that compress surrounding structures [14]. The secondary phase, which can last for days, includes hematoma expansion and cerebral edema, resulting from blood–brain barrier (BBB) breakdown in the tissue surrounding the clot [15, 16] and a prominent inflammatory response with the infiltration of neutrophils and monocytes, activation of resident microglia, and cross talk with other brain cells. These events often occur after hospital admission and are therefore temporally amenable to therapeutic intervention [17–19]. Many studies have focused on the initial capacity of the activated microglia to promote bystander injury; however, the innate immune response in the CNS, as in peripheral tissues, can orchestrate the repair, reconstruction, and resolution of tissue injury. There is a need for studies of the evolution of the inflammatory response over time after ICH and comparisons between young adult and aged animals.

We used real-time RT-PCR to compare the expression of 27 immune-related molecules in young and aged rats at 6 h and 1, 3, and 7 days after ICH was induced by injecting bacterial collagenase into the striatum, a model we have previously used to study inflammation and damage to gray and white matter [11, 12, 20–23]. Our salient earlier findings (illustrated in the summary image in Electronic Supplementary Material (ESM) Fig. S1) are as follows. Neutrophils infiltrate the hematoma only and at early times (1 and 3 days) and then die off. By day 3, there is extensive neuron death, damage to myelin and axons, and infiltration of the damaged axon bundles by activated microglia and macrophages within and at the edge of the hematoma. Over the first several days, the hematoma becomes filled with activated microglia and macrophages, and a glial scar forms at the lesion edge, with a band of activated microglia and macrophages surrounded by reactive astrocytes. Therefore, we selected this time course to analyze the expression of inflammation-related genes, including pro- and anti-inflammatory mediators, tissue remodeling enzymes, and markers of different states of macrophage/microglia activation.

## Materials and Methods

### Induction of Intracerebral Hemorrhage

All procedures were performed on male Sprague–Dawley rats in accordance with the guidelines established by the Canadian Council on Animal Care and approved by the Animal Care Committee at the University Health Network. Young adult rats (*n* = 36) were 3–4 months old and 350–500 g at the time of surgery (Charles River, St-Constant, Canada). Aged rats (*n* = 35) were purchased at 10–11 months old from Harlan Laboratories (Mississauga, Canada) and maintained in our animal facility until 20–21 months old and 450–600 g. With a mean life span of ~25 months for this rat strain [24], this corresponds to early old age in humans. ICH was induced in the anterior striatum (caudate + putamen), as we recently described [12, 20]. In brief, rats were anesthetized with isoflurane (3.5% induction, 1.5% maintenance), placed in a small-animal stereotaxic instrument (David Kopf Instruments, Tujunga, CA), and a 1-mm diameter burr hole was drilled 0.2 mm anterior and 3.5 mm lateral to bregma. A 30-gauge needle attached to a micropump (UltraMicroPump II, World Precision Instruments, Sarasota, FL) was lowered 6 mm ventral to the skull surface into the right caudate putamen.

**Fig. 1:**
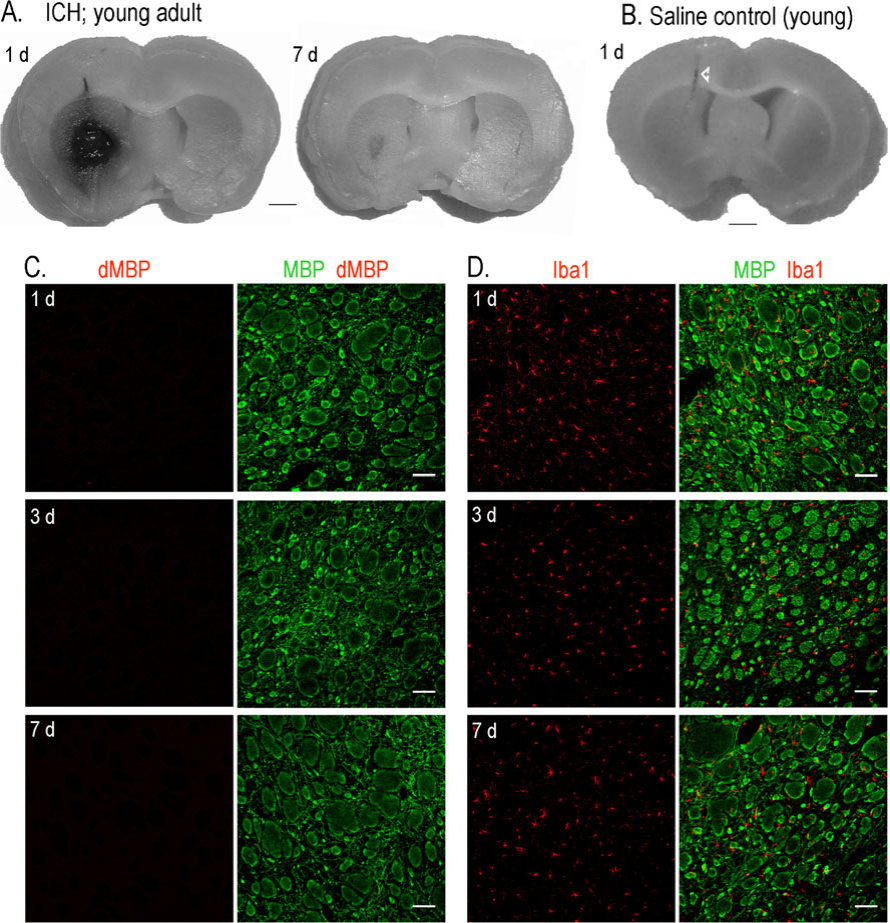
The collagenase model of ICH and the use of the contralateral striatum for animal- and age-matched comparisons. **a** A representative young adult rat showing that stereotaxic injection of type IV collagenase produced a hematoma that was restricted to the ipsilateral striatum. Much of the blood clot has been resorbed by day 7. **b** In a saline-injected control rat, the needle penetration track is seen (*arrowhead*) without bleeding in the striatum. **c** Representative confocal images of the contralateral striatum at 1, 3, and 7 days after ICH show normal white matter bundles labeled with myelin basic protein (*MBP*, *green*) and lack of degraded MBP (*red*). **d** In the contralateral striatum at 1, 3, and 7 days after ICH, microglia, identified with Iba1 (*red*) are of ramified morphology and broadly distributed between the white matter tracts. *Scale bars*, 500 μm

ICH was evoked by injecting bacterial type IV collagenase (Sigma-Aldrich, Oakville, Canada) to rupture blood vessels. This is a well-characterized model [25–28] that we have used extensively [11, 12, 20–23] (and see ESM Fig. S1). Collagenase was diluted in sterile physiological saline, and the injected amounts (0.2 U for young adult rats, 0.15 U for aged rats; both in 0.5 μL saline) were chosen to produce lesions that were limited to the striatum (examples shown in Fig. 1a). Using the lower dose in aged rats minimized mortality and created lesion sizes that were comparable to those in young adult rats [11]. Note that lesion size cannot be measured in the same animals used for real-time RT-PCR because the whole striatum is rapidly harvested to preserve the mRNA. However, visual inspection of the hematoma before harvest did not identify apparent differences in size between young and aged animals. Control animals were time- and age-matched sham-operated rats that received intrastriatal injections of 0.5 μL saline. In all cases, the injection rate was 0.25 μL/min, after which the needle was left in place for 5 min to prevent solution reflux. Core body temperature was maintained at 36.5°C throughout the surgery and recovery period using a thermostat-regulated heating pad. All rats survived and regained consciousness within 10 min. As expected, all rats with an ICH (but not saline-injected control rats) demonstrated an ipsilateral turning bias, while their grooming and feeding behavior appeared normal.

### Tissue Preparation

Cohorts of animals were killed by an overdose of isoflurane at 6 h and 1, 3, or 7 days after surgery. For real-time RT-PCR, animals were perfused transcardially with 120 mL of cold PBS (0.1 mol/L phosphate-buffered saline, pH 7.5). Brains were removed, placed in a rat brain matrix (Ted Pella Inc., Redding, CA), submerged in ice-cold PBS, and cut 2 mm anterior and 2 mm posterior to the needle entry site, which was easily identified on the brain surface. Each 4-mm-thick coronal section was divided along the midline into an ipsilateral (hematoma-bearing) and contralateral hemisphere, and the striatum was isolated from each slice and snap-frozen on dry ice. Tissue was stored at −80°C until used for RNA extraction. For immunohistochemistry, animals were perfused transcardially with PBS (as above), followed by fixative (120 mL cold PBS-buffered 4% paraformaldehyde; EMD Biosciences, Gibbstown, NJ). Brains were removed and processed at 4°C: first in fixative (24 h), then in 10% sucrose in PBS (24 h), and finally in 30% sucrose in PBS until the brains sank. The cryoprotected brains were placed in a brain matrix; coronal slices were made at 3, 6, and 9 mm from the anterior and then mounted in Tissue Freezing Medium (Somagen Diagnostics, Edmonton, Canada) and stored at −40°C. Frozen brain sections (16 μm thick) were made on a cryostat (model CM350S, Leica, Richmond Hill, Canada), collected on gelatin-coated slides (1% gelatin, 0.5% chromium potassium sulfate), and stored at −40°C until used.

### Quantitative Real-Time Reverse Transcriptase Polymerase Chain Reaction

**Fig. 2:**
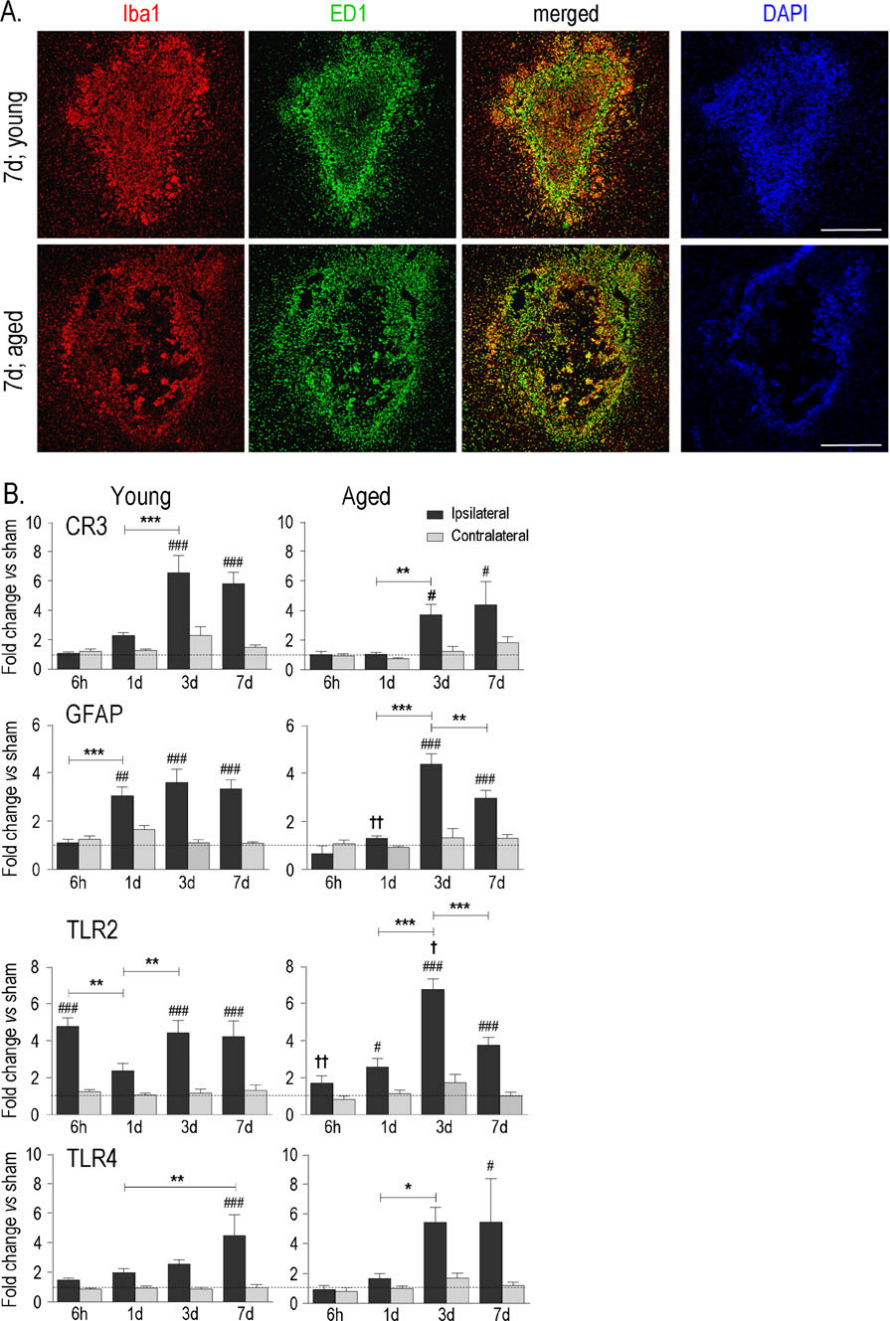
Time-dependent glial cell accumulation and activation. **a** Low magnification coronal images of the hematoma at 7 days post-ICH in representative young adult and aged rats. Microglia and macrophages (*red*) were labeled with Iba1 (*red*) and the lysosomal marker, ED1 (*green*). Adjacent serial sections were stained with the nuclear dye, DAPI (*blue*). *Scale bars*, 500 μm. **b** Expression of several markers of glial responses after ICH: astrocyte marker, glial fibrillary acidic protein (*GFAP*); microglia/macrophage markers, complement receptor 3 (*CR3*), Toll-like receptors, TLR2 and TLR4. After two-way ANOVA with Bonferroni correction, the following significant intergroup differences are indicated: ^#^In ipsilateral vs. contralateral striatum; *With time; ^†^Between young adult and aged rats. One symbol of any type signifies *p* < 0.05; two symbols, *p* < 0.01; three symbols, *p* < 0.001

**Fig. 3:**
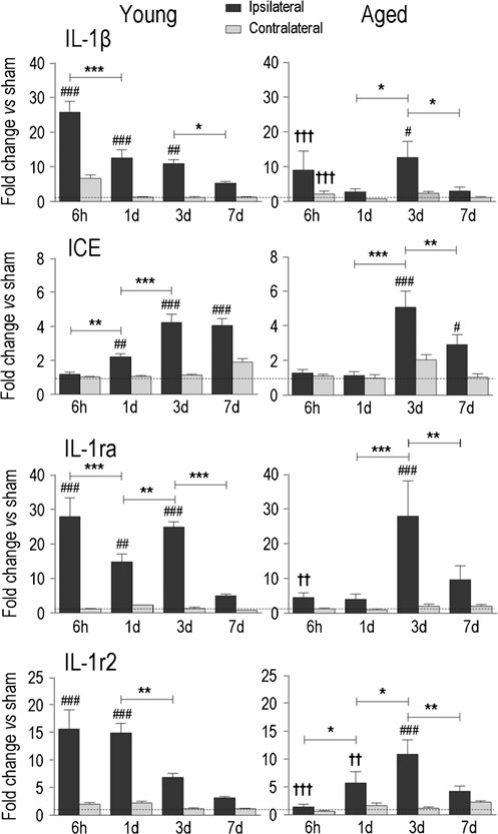
Temporal expression of IL-1β and its regulatory molecules in young adult and aged rats after ICH. Transcript expression was measured, analyzed, and expressed as in Fig. 2b for interleukin-1β (*IL-1*β), interleukin-1-converting enzyme (*ICE*), the endogenous IL-1 receptor antagonist (*IL-1ra*), and type 2 IL-1 receptor (*IL-1r2*)

**Fig. 4:**
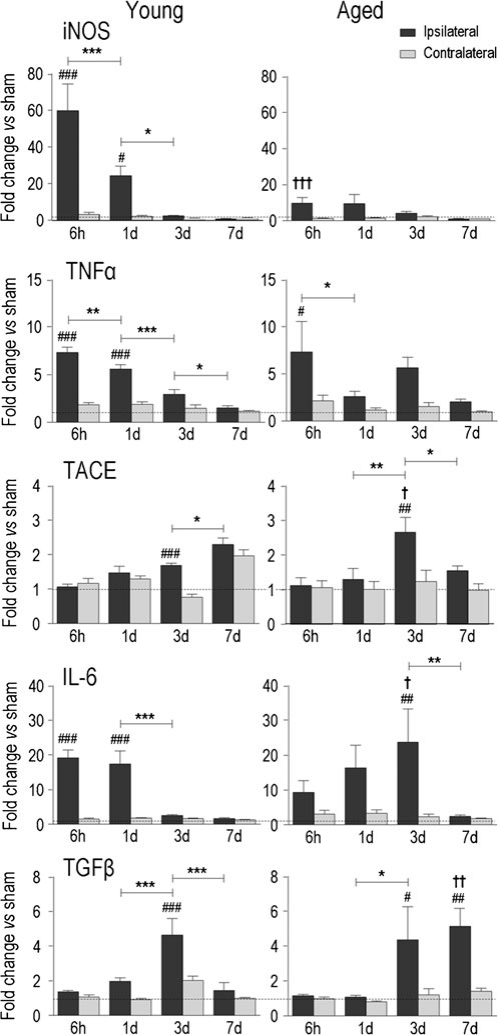
Temporal expression of hallmark inflammatory molecules after ICH in young adult and aged rats. Transcript expression was measured, analyzed, and expressed as in Fig. 2b for inducible nitric oxide synthase (*iNOS*/*NOS*_*2*_), tumor necrosis factor-α (*TNF*α), TNFα-converting enzyme (*TACE*), interleukin-6 (*IL-6*), and transforming growth factor-β (*TGF*β)

**Fig. 5:**
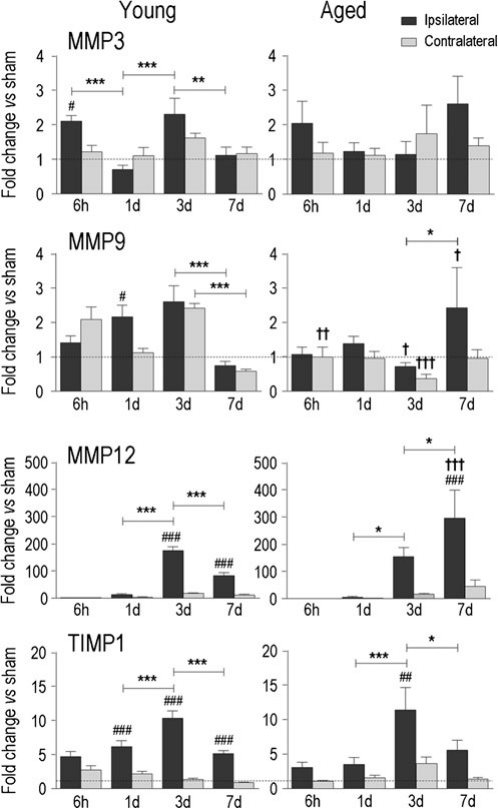
Temporal expression of matrix metalloprotease-related genes after IHC in young adult and aged rats. Transcript expression was measured, analyzed, and expressed as in Fig. 2b for the matrix metalloproteases, MMP3, MMP9, MMP12, and the endogenous tissue inhibitor of metalloproteinases (*TIMP1*)

**Fig. 6:**
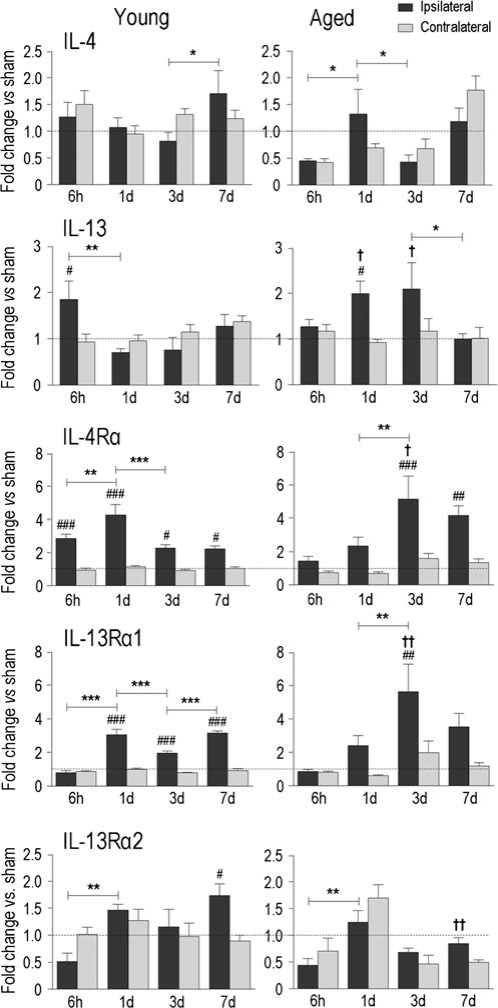
Temporal expression in young adult and aged rats of genes involved in alternative macrophage/microglia activation. Transcript expression was measured, analyzed, and expressed as in Fig. 2b for interleukin-4 (*IL-4*), interleukin-13 (*IL-13*), and their receptors, IL-4Rα, IL-13Rα1, IL-13Rα2

**Fig. 7:**
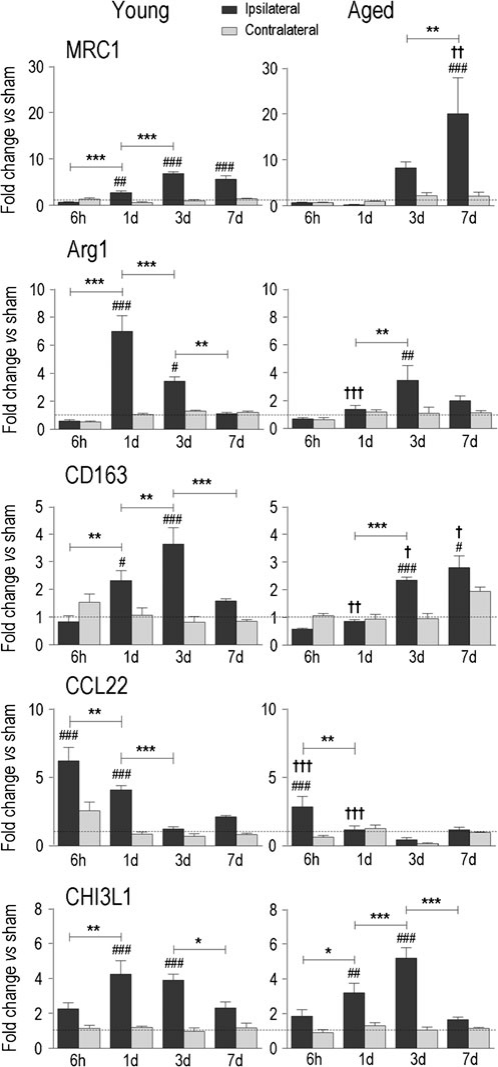
Temporal expression of markers of alternative activation in young adult and aged rats. Transcript expression was measured, analyzed, and expressed as in Fig. 2b for mannose receptor-1 (*MRC1*), arginase 1 (*ARG1*), the haptoglobin–hemoglobin scavenger receptor (*CD163*), chemokine (*C–C motif*) ligand 22 (*CCL22*), and chitinase 3-like 1 (*CHI3L1*)

Gene-specific primers (Table 1) were designed using “Primer3Output” (http://frodo.wi.mit.edu/cgi-bin/primer3/primer3_www.cgi). Total RNA was extracted using the TRIzol method (Invitrogen), followed by further purification using the RNeasy Mini Kit (Qiagen, Mississauga, ON), and a two-step reaction was performed according to the manufacturer’s instructions (Invitrogen). In brief, total RNA (0.8 μg) was reverse-transcribed in 20 μL volume using 200 U of SuperScriptII RNase reverse transcriptase, with 0.5 mM dNTPs and 0.5 μM oligo dT (Invitrogen). Amplification was performed on an ABI PRISM 7900 Sequence Detection System (PE Biosystems, Foster City, CA) at 95°C for 10 min, 40 cycles at 95°C for 15 s, and 56°C for 20 s. “No-template” and “no-amplification” controls were included for each gene, and melt curves showed a single peak, confirming specific amplification. The threshold cycle (*C*_T_) for each gene was normalized to that of the housekeeping gene, hypoxanthine guanine phosphoribosyl transferase (HPRT1), which we have found to be especially stable in the brain. Results in Figs. 2, 3, 4, 5, 6, and 7 are mRNA expression relative to the level in control rats injected with saline (mean ± SEM, *n* = 4–6 rats per age group per time point). For statistical analysis, two-way ANOVA and Bonferroni post hoc tests were conducted using GraphPad Prism ver 5.01 (GraphPad Software, San Diego, CA).

**Table 1:** Primers used for real-time RT-PCR

Gene	GenBank accession no.	Primer sequences
ARG1	NM_017134.1	FP: GTCTGTGGGAAAAGCCAATG
		RP: TTGCCATACTGTGGTCTCCA
CR3	NM_012711	FP: TGCTGAGACTGGAGGCAAC
		RP: CTCCCCAGCATCCTTGTTT
CCL22	NM_057203.1	FP: AGGATGCTCTGGGTGAAGAA
		RP: TAGGGTTTGCTGAGCCTTGT
CD163	NM_001107887.1	FP: ATCACAGCATGGCACAGGT
		RP: TCCAGATCATCCGTCTTCG
CHI3L1	NM_053560	FP: GGGCAGTGGATTTGGATG
		RP: TGCAAGTGACCAGACTCCTG
GFAP	NM_017009.1	FP: CAGCTTCGAGCCAAGGAG
		RP: TGTCCCTCTCCACCTCCA
ICE	NM_012762	FP: CCAACCACTGAAAGGGTGA
		RP: GCATGATTCCCAACACAGG
IL-1β	NM_031512	FP: TGACCCATGTGAGCTGAAAG
		RP: AGGGATTTTGTCGTTGCTTG
IL-1ra/IL-1rn	NM_022194	FP: GGGAAAAGACCCTGCAAGA
		RP: GTGGATGCCCAAGAACACA
IL-1r2	NM_053953.1	FP: GTGATCATTTCTCCCCTGGA
		RP: CACGATGGTGTTGGAAGATG
IL-6	NM_012589	FP: CAGGAACGAAAGTCAACTCCA
		RP: ATCAGTCCCAAGAAGGCAACT
IL-4	NM_201270.1	FP: CAAGGAACACCACGGAGAA
		RP: TTCAGACCGCTGACACCTC
IL-4rα	NM_133380.2	FP: TCCGCACTTCTACGTGTGAG
		RP: AGACCACAGTTCCAGCCAGT
IL-13	NM_053828.1	FP: TCTGTGCAGCCCTGGAAT
		RP: GCGGAAAAGTTGCTTGGA
IL-13rα1	NM_145789.2	FP: AGAAACATGGAGGGTGCAAG
		RP: CACTGCGACAAAGACTGGAA
IL-13rα2	NM_145789.2	FP: AGAAACATGGAGGGTGCAAG
		RP: CACTGCGACAAAGACTGGAA
iNOS	NM_012611	FP: GCTACGCCTTCAACACCAA
		RP: GCTTGTAACCACCAGCAGT
MRC1	NM_001106123	FP: AAGGTTCCGGTTTGTGGAG
		RP: TGCATTGCCCAGTAAGGAG
MMP-3	NM_133523	FP: TCCCACAGAATCCCCTGA
		RP: CGCCAAAAGTGCCTGTCT
MMP-9	NM_031055	FP: CTGCCTGCACCACTAAAGG
		RP: GAAGACGAAGGGGAAGACG
MMP-12	NM_053963	FP: CTGGGCAACTGGACACCT
		RP: CTACATCCGCACGCTTCA
TIMP1	NM_053819	FP: GGTTCCCCAGAAATCATCG
		RP: GGAAACCTGTGGCATTTCC
TACE	NM_020306	FP: TGTGAGCAGTTTCTCGAACG
		RP: AAAGGCACCAAAGTGGTCAG
TGFβ1	NM_021578.1	FP: ATACGCCTGAGTGGCTGTC
		RP: GCCCTGTATTCCGTCTCCT
TLR2	NM_198769.2	FP: CTGCAAGCTCTTTGGCTCTT
		RP: ACACACCAGCAGCATCACAT
TLR4	NM_019178.1	FP: TGCTCAGACATGGCAGTTTC
		RP: GCGATACAATTCGACCTGCT
TNF-α	NM_012675	FP: GCCCACGTCGTAGCAAAC
		RP: GCAGCCTTGTCCCTTGAA
HPRT-1	NM_012583.2	FP: CAGTACAGCCCCAAAATGGT
		RP: CAAGGGCATATCCAACAACA

*FP* forward primer, *RP* reverse primer

### Immunohistochemistry

Frozen brain sections mounted on slides were thawed at room temperature and rehydrated for 20 min in PBT buffer (0.1 mol/L PBS, pH 7.5, 0.1% bovine serum albumin, 0.2% Triton X-100). To reduce nonspecific antibody binding, slides were blocked for 2 h in 10% donkey serum in PBT. Brain sections were then incubated overnight at room temperature in PBT buffer that contained one or two of the following primary antibodies. Axon bundles were labeled with a mouse monoclonal antibody against myelin basic protein (MBP; 1:100; Sigma-Aldrich), and regions of myelin damage or demyelination were identified with a rabbit polyclonal antibody that recognizes only degraded MBP (dMBP, 1:250; Chemicon, Temecula, CA). The microglia were labeled with rabbit polyclonal anti-ionized calcium-binding adapter-1 (Iba1; 1:1,000; Wako, Japan) or a mouse monoclonal anti-ED1 antibody (1:100; Serotec, Raleigh, NC) which labels CD68 in the lysosomes of activated phagocytic microglia/macrophages. Microvessels were labeled with a rabbit polyclonal antibody against collagen type IV (CgIV; 1:250; Abcam, Cambridge, MA), a major constituent of the basal lamina in cerebral blood vessels [29]. After labeling with primary antibodies, brain sections were washed in PBS (3 × 10 min) and incubated for 2 h at room temperature in the dark with appropriate secondary antibodies diluted to 1:400 in PBT, i.e., DyLightTM488-conjugated AffiniPure donkey anti-mouse IgG, DyLightTM594-conjugated AffiniPure donkey anti-rabbit or anti-mouse (Jackson Immunoresearch, Mississauga, Canada). To stain cell nuclei, 4′-6-diamidino-2-phenylindole (DAPI; 1:5,000; Sigma-Aldrich) was applied for 5 min. After washing in PBT (3 × 20 min), slides were coverslipped using Vectashield mounting medium (Cedarlane, Burlington, Canada) and stored in the dark at 4°C. All images shown are representative examples of at least three animals at each indicated time point.

## Results

Because ICH commonly occurs in deep nuclei of the cerebrum, pons, cerebellum, and in deep white matter [2], we have been studying the anterior striatum of the rat (caudate and putamen) [11, 12, 20–23]. After collagenase is injected into the striatum, a hematoma rapidly develops and then the blood clot is gradually resorbed over several days (Fig. 1a), leaving a lesion with neuron death, white matter damage, BBB disruption, and extensive accumulation of the activated microglia/macrophages (the collective term reflects that the cells become morphologically indistinguishable after microglia processes retract). ESM Fig. S1 shows examples of immunostaining to illustrate the temporal–spatial evolution of the ICH lesion. In saline-injected control rats (Fig. 1b), the needle track can be seen, but there is no bleeding in the striatum. The contralateral striatum was uninjured (Fig. 1c, d); it showed normal MBP staining of white matter tracts (bundles of penetrating axons in cross-section), no dMBP, and normal microglia with a “resting” ramified morphology. Therefore, over the 7-day time course of the present study, we routinely compared the damaged ipsilateral striatum with saline-injected control animals and used the contralateral striatum as an animal- and time-matched internal reference [20, 23]. Importantly, the collagenase dose was titrated to obtain comparable sized lesions in young and aged rats (examples in Fig. 2a) [11].

### Time-Dependent Glial Cell Activation in Young and Aged Rats

The inflammatory response after ICH is characterized by neutrophil and monocyte infiltration, activation of endogenous microglia and astrocytes, and production of numerous inflammatory mediators, including cytokines and matrix metalloproteases (MMPs). We previously quantified the time-dependent accumulation of activated microglia/macrophages over the first week in this ICH model [12, 20, 22] (and see ESM Fig. S1). In young rats, most of the blood clot was resorbed by 7 days (Fig. 1a), presumably due to phagocytosis. Interestingly, activated microglia/macrophages in the lesion were not homogeneous (Fig. 2a); Iba1-labeled cells filled the lesion, while those labeled with the lysosomal marker, ED1 (an indicator of phagocytic capacity) were more concentrated at the periphery, where we previously showed a zone of dramatic neuron loss [11, 22] (and see ESM Fig. S1). Despite ensuring initially similar-sized hematomas in young adult and aged rats, by 7 days the lesion of aged rats showed less Iba1 labeling and fewer total cell numbers (nuclei stained with DAPI). ED1 staining was more diffuse and extended further into the surrounding striatum. Based on these results and previously reported differences between young and aged rats (see “Discussion”), we examined the time course (6 h, 1, 3, and 7 days) of induction of 27 genes associated with inflammation.

We assessed gene transcript expression in each ipsilateral and contralateral striatum (four to six rats per group). In order to identify ICH-induced changes, values on the *Y*-axes (Figs. 2, 3, 4, 5, 6, and 7) indicate fold changes normalized to age- and time-matched, saline-injected control animals (set to 1.0, dashed line in each panel). Because increased basal levels of cytokines (TNFα, IL-1α, IL-1β, IL-6, TGFβ) have been reported in the aging naive rodent brain [30–32], we have not compared basal levels in young and aged control animals.

First, we monitored several molecules that are commonly used to assess gliosis and inflammation after acute brain injury [33–35], i.e., the astrocyte marker, glial fibrillary acidic protein (GFAP), and the microglia/macrophage markers, complement receptor 3 (CR3/CD11b) and Toll-like receptors, TLR2 and TLR4. Over the first week after ICH, GFAP and CR3 increased in the damaged ipsilateral striatum of young rats (Fig. 2b) and reached levels several-fold higher than saline-injected controls. The main difference in aged rats was the lack of up-regulation of GFAP at 1 day, which might reflect slower development of a glial scar, and correlates with our earlier finding that white matter injury extends further in aged rats [12]. While CR3 was suggestive of a lower expression in aged animals, more data would be needed. The up-regulation of TLR2 and TLR4 expression further indicates an innate immune response, but interestingly, in young animals, TLR2 clearly preceded TLR4 induction (6 h vs. 7 days). Activation of microglia is thought to depend on the TLR2/TLR4 activation sequence, with TLR2 activation decreasing TLR4 transcription and downstream expression of several phagocytosis-related receptors (CR3, MHCII, FcyR) [36]. In aged animals, TLR2 and TLR4 showed a delay and a similar time course, with the highest expression on days 3 and 7 (none of the four genes was up-regulated in the contralateral striatum).

### Time- and Age-Dependent Induction of Pro-inflammatory Genes and MMPs

The pro-inflammatory cytokine, IL-1β, is one of the earliest mediators induced and is considered a principal orchestrator of inflammation after acute CNS injury [37]. IL-1β function requires interleukin-1 converting enzyme (ICE/caspase 1) which cleaves pro-IL-1β to its mature, active form [38, 39]. The bioavailability of IL-1 is reduced by specific decoy receptors expressed on the macrophage/microglia surface or secreted into the local environment, i.e., the IL-1 receptor antagonist (IL-1ra), which binds to the receptors without inducing a cellular response [40], and the type 2 IL-1 receptor (IL-1r2), which acts as a decoy receptor [41]. In young animals, there was a marked and rapid IL-1β induction after ICH (~25-fold increase over controls by 6 h), after which the expression progressively declined (Fig. 3). ICE up-regulation was lower (at most ~4-fold) and lagged behind, increasing from 1 to 3 days. In aged animals, IL-1β was lower (~10-fold increase at 6 h), and ICE induction was delayed until day 3. Striking age-related differences were seen for the endogenous antagonists, IL-1ra and IL-1r2. Both showed a dramatic and early induction in young rats; for instance, at 6 h, the increases were ~27-fold for IL-1ra and ~15-fold for IL-1r2. In aged rats, neither antagonist was significantly elevated until 3 days, which suggests that functionally active IL-1β might be elevated in the aged (NB: In the contralateral striatum, there was no induction of ICE, IL-1ra or IL-1r2, while IL-1β was increased slightly at 6 h in young animals).

Inducible nitric oxide synthase (iNOS/NOS_2_) produces nitric oxide which can contribute to the neurotoxic capacity of activated microglia and macrophages [42–45]. A striking age-related difference in iNOS induction was seen (Fig. 4). In young adult rats, iNOS expression increased rapidly and dramatically after ICH (~60-fold at 6 h) and then declined over the next 3 days. In contrast, induction in the aged (ipsilateral vs. contralateral) was not statistically significant at any time examined. Tumor necrosis factor-α (TNFα) is an early orchestrator of the inflammatory response after acute brain injury [46] and is activated by the TNFα-converting enzyme, TACE/ADAM17 [47]. In young rats, TNFα induction followed a similar trend to IL-1β: rapid up-regulation (6 h and 1 day) and a time-dependent decline. In aged animals, TNFα was increased only at 6 h. Interestingly, TACE was only increased at 3 days and reached a higher level in aged animals. Interleukin-6 (IL-6) is a pleiotropic cytokine, and its expression is regulated by the classical IL-1 pathway [48]. In young rats, IL-6 was markedly elevated early after ICH (15- to 20-fold higher than controls at 6 h and 1 days) and then declined to baseline by 3 days. In aged rats, IL-6 induction showed a gradual increase and peaked at 3 days. Transforming growth factor-β (TGFβ) is thought to promote the damage resolution phase after acute CNS injuries [49], including glial scar formation [50]. In young rats, TGFβ increased transiently at 3 days, but elevation was more prolonged in aged rats (3 and 7 days).

MMPs contribute to extracellular matrix remodeling and cleavage of precursor molecules, but their elevation after ICH also correlates with blood BBB disruption, hematoma expansion, and edema (reviewed in [51]). There were marked increases in MMP12 in both age groups (150- to 200-fold over control rats), which peaked at 3 days in the young but continued to increase at 7 days in the aged animals (Fig. 5). In contrast, there was little change in MMP3 and MMP9 in the young rats (at most a 2- to 2.5-fold increase over control rats), and they were not up-regulated in aged rats. After a slight increase in MMP3 at 6 h in the young animals, an apparent oscillation with time (lower at 1 and 7 days) did not significantly differ from controls. The small changes in MMP9 were difficult to interpret because the levels apparently increased and oscillated in the contralateral striatum. The endogenous tissue inhibitor of matrix metalloproteases (TIMP1), which regulates the activity of all three MMPs, was elevated on days 1, 3, and 7 in the young, but only on day 3 in aged rats.

### Time-and Age-Dependent Induction of Resolving and Alternative Activation Genes

The initial pro-inflammatory phase after acute injury is characterized by classical (also called M1) activation of macrophages and microglia. This is followed by resolution of the defense response and a repair phase, with changes in the state of macrophages/microglia to what is variously called M2, alternative activation, or acquired deactivation, states that are most readily defined by stimuli and outcomes in vitro (reviewed in [52, 53]). While recognizing that multiple stimuli are present after acute damage in vivo, for simplicity, we will use the term alternative activation when describing resolving and repair genes. The observed spatial differences in Iba1 and ED1 expression in activated microglia/macrophages (Fig. 2a), and differing temporal expression of TLR2 and TLR4 between young and aged rats (Fig. 2b), point to a complexity in their activation states after ICH. We first analyzed changes in the expression of two known inducers of alternative macrophage/microglia activation (IL-4 and IL-13) and the three receptors through which they act: IL-4Rα, IL-13Rα, and IL-13Rα2 (Fig. 6). IL-13, IL-4Rα, and IL-13Rα1 increased above control levels, and this occurred later in aged rats, i.e., IL-4Rα at 1 and 3 days in young vs. 3 and 7 days in aged; IL-13Rα1 at 1, 3, and 7 days in young vs. 3 days only in aged; IL-13 at 6 h in young vs. 1 and 3 days in aged rats. IL-4 was not significantly increased above control levels and instead was apparently suppressed early after ICH in aged rats (6 h). IL-13Rα2 only increased above control levels in the young and only at 7 days.

**Fig. 8:**
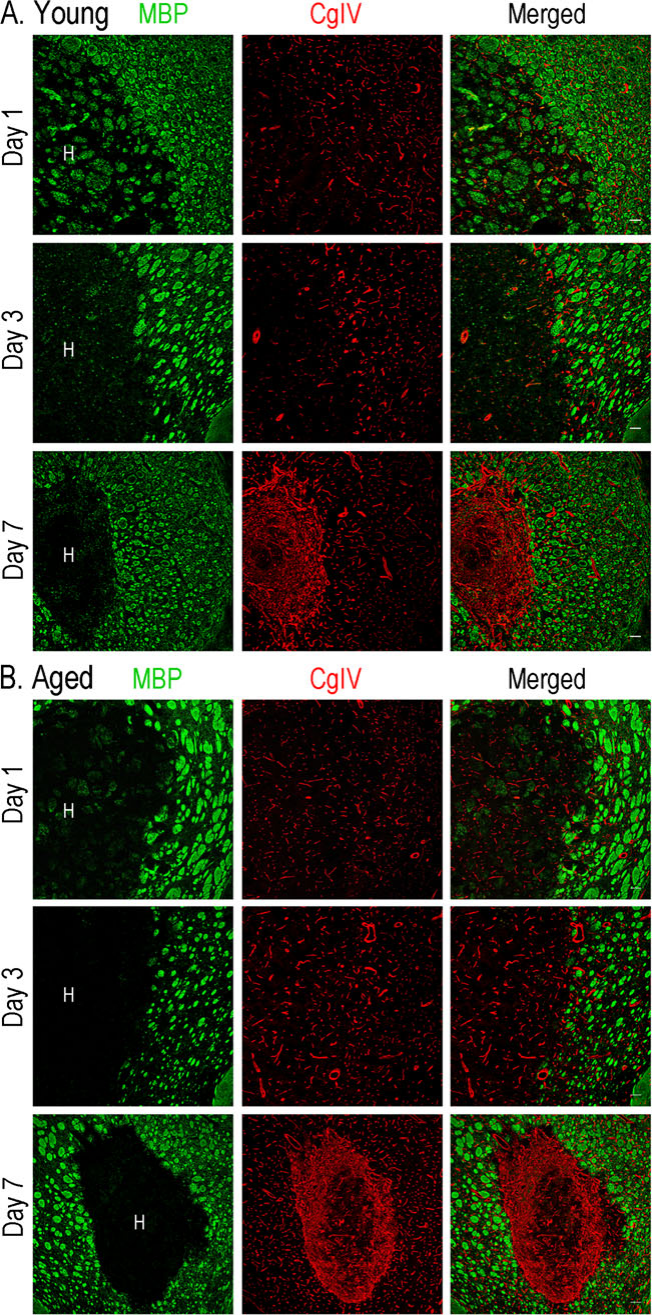
Distribution of collagen type IV (*CgIV*) staining after ICH. Representative confocal images of young (**a**) and aged (**b**) rats show myelin basic protein (*MBP*, *green*) labeling of white matter tracts and CgIV staining (*red*). The hematoma (*H*) is readily seen as loss of MBP staining. At 1 and 3 days, CgIV localized to blood microvessels. At 7 days, the staining intensity greatly increased inside the lesion. *Scale bars*, 100 μm

Next, we monitored the expression of several well-known markers of alternatively activated macrophages/microglia: mannose receptor (MRC1/CD206), arginase 1 (ARG1), haptoglobin–hemoglobin scavenger receptor (CD163), and chemokine (C-C motif) ligand 22 (CCL22). In young adult rats, all four markers rapidly increased: by 6 h for CCL22 and by 1 day for the others. MRC1 and CD163 increased further with time, and while MRC1 was sustained until 7 days, the other genes decreased. There were several notable differences in the aged rats: MRC1 was induced later, but was higher at 7 days; ARG1 was lower at 1 day; CD163 was lower at 1 and 3 days, but higher at 7 days; and CCL22 was lower at 6 h and 1 day. Together, these results suggest that alternative activation was delayed in the aged animals. Because ARG1 metabolizes l-arginine to produce l-ornithine, which can be further metabolized into l-proline, a substrate for collagen synthesis [54], we examined the basal lamina component, collagen type IV (CgIV; Fig. 8). At 1 and 3 days after ICH, CgIV localized to blood microvessels, including fragmented vessels inside the hematoma. At 1 day, some white matter bundles inside the hematoma still stain for myelin basic protein, but they are swollen and disrupted, as we have previously shown [20]. Surprisingly, at 7 days, in addition to pronounced microvessel staining around the lesion, the core was filled with a more diffuse deposit of CgIV that appeared as a fibrotic clot. This response in the core was evident at 7 days in both young and aged animals, and there was no apparent difference in the time course of cgIV accumulation. Not all genes associated with alternative activation differed with age, e.g., chitinase 3-like 1 (CHI3L1), which can also induce alternative macrophage activation [55], was up-regulated at 1 and 3 days in both age groups.

## Discussion

Despite the evidence that inflammation plays a crucial role in ICH pathology, surprisingly little is known about the timing of molecular changes, especially in aged rats. A microarray study on humans [56] and two on young adult rats [57, 58] examined a single time point (24 h) and noted changes in many immune-related molecules, including IL-1β, IL-1 receptor type 2, IL-6 receptor, TGFβ, TLR1, TNF receptor, IL-13Rα1, IL-10 receptor β, interferon receptor 2, macrophage inflammatory protein-2 precursor, prepro- complement C3, and MHC class II RT1-B region Ia antigen. Temporal changes in young rodents are beginning to be examined, but have been limited to a few pro-inflammatory (C3, CR2, IL-1β, IL-6, interferon-γ, NF-κB, TNFα) and inflammation-resolving molecules (IL-10, TGFβ) [59, 60]. We previously analyzed several inflammatory genes (CR3, ICE, TACE, IL-1ra) and MMPs (MMP3, MMP9, MMP12) following ICH in young rats [21, 23], and the recent study on mice [59] is in good agreement with our results. The present study is unique in that it analyzes: (1) 27 genes selected to encompass a broad spectrum of inflammation-related molecules, including pro- and anti-inflammatory mediators, tissue remodeling enzymes, and markers of different states of macrophage/microglia activation; (2) four time points (6 h and 1, 3, and 7 days) that bracket the secondary injury phase; (3) the ipsilateral and contralateral striatum, after normalizing to time-matched saline-injected control rats; and (4) young adult and aged rats at each time point.

Several cell types have the potential to produce inflammatory mediators after brain injury, including endogenous microglia and astrocytes, and infiltrating neutrophils and macrophages [61, 62]. Before discussing changes in gene expression, it is worth considering cellular responses, which have been mainly studied in young animals (reviewed in [63, 64]). Neutrophils begin to infiltrate as early as 4 h [65] and are an important source of TNFα and IL-1β [66], which help recruit blood monocytes/macrophages [67]. Early microglial activation can occur in the peri-hematomal region [63, 68, 69]. In the ICH model used in this study (see ESM Fig. S1), we have observed a pronounced inflammatory response with rapid infiltration of neutrophils (within hours) and later activation of microglia and astrocytes, infiltration of blood monocytes/macrophages, and white matter damage. By 3 days after ICH induction, activated microglia, macrophages, and astrocytes formed a glial scar surrounding the hematoma. Neutrophil depletion reduced the activation of microglia, macrophages, and astrocytes and decreased BBB breakdown and axon damage [20]. Here, as early as 6 h after ICH in young adult rats, we observed increases in the expression of TLR2, IL-1β, IL-1ra, IL-1r2, iNOS, TNFα, IL-6, MMP3, IL-13, IL-4Rα, and CCL22. These increases are too early for macrophage infiltration [70] and might be due to neutrophil infiltration (e.g., TNFα was mainly in neutrophils early after ICH [20, 21]) or the responses of endogenous brain cells, including microglia, to blood products. Of note is that thrombin is produced in the brain immediately after ICH, and it can induce microglia to secrete pro-inflammatory molecules (IL-1β, TNFα) [71] and chemotactic factors that recruit blood leukocytes [72].

There are reports of poorer functional recovery after ICH in aged animals [10, 13, 73] and histological differences, including reduced lesion resolution [11], more white matter damage [12], more severe edema and autophagy [10, 13], and decreased synaptogenesis and angiogenesis [74]. All of these factors likely contribute to poorer functional outcomes, but how might the inflammatory response contribute? Reduced neutrophil infiltration has been reported in aged rats after ICH [75], and we observed a more widespread distribution of ED1-labeled phagocytic microglia/macrophages [11] (present study), especially in damaged white matter bundles [20]. Here, we found differences between young adult and aged rats in induced levels and/or timing for 18 of the 27 genes examined: TLR2, GFAP, IL-1β, IL-1ra, IL-1r2, iNOS, IL-6, TGFβ, MMP9, MMP12, IL-13, IL-4Rα, IL-13Rα1, IL-13Rα2, MRC1, ARG1, CD163, CCL22. Most were delayed induction or lower levels in aged rats, with several differing as early as 6 h (TLR2, IL-1β, IL-1ra, IL-1r2, iNOS, CCL22), and thus likely reflecting changes in neutrophil infiltration and dampened responses of endogenous brain cells. Given the many changes observed, rather than discussing every gene, we will focus on functional groupings, as in “Results.“

As markers of the glial response, the induction of GFAP and TLR2 was delayed in aged rats, CR3/CD11b up-regulation was reduced, and, at 7 days, ED1-labeled activated microglia/macrophages were more diffusely distributed in the surrounding striatum. IL-1β is a principal orchestrator of brain inflammation [37] which contributes to activating microglia [48], astrocytes [76–78], and endothelial cells [62] that then secrete IL-1β and other inflammatory molecules. IL-1β has been the focus of many experimental studies of ischemic stroke [79]. Intracerebroventricular or stereotaxic striatal injection of exogenous IL-1β exacerbated stroke-induced damage in young animals [80], and conversely, injury was reduced after the IL-1β antagonist, IL-1ra, was injected either locally into the striatum [81] or into an intracerebral ventricle [82]. Here, we found that early after ICH, young rats had a higher expression of IL-1β and its regulatory molecules, IL-1ra and IL-1r2, possibly as a compensatory response, as well as higher iNOS and IL-6 levels. In aged rats, the increase in IL-6 was delayed and TGFβ elevation was prolonged. Our results demonstrate the complexity and differences between young adult and aged rats in mediators generally associated with classical (or M1) macrophage/microglia activation.

In order to restore tissue homeostasis, it is expected that macrophage/microglia activation will shift from a pro-inflammatory gene profile to one that supports repair and tissue reconstruction. In peripheral tissue, this switch is induced primarily by the anti-inflammatory cytokines, IL-4, IL-13, IL-10, and TGFβ (reviewed in [52, 53, 83, 84]). Interestingly, IL-4 knockout mice had a larger infarct volume and poorer neurological outcome after ischemic stroke [85]. In this first study of alternative activation markers after ICH or in aged animals, we observed the induction of IL-13 (by 1 day) and TGFβ (by 3 days) and a delay in aged rats. We previously showed IL-10 induction at 6 h and 7 days in young rats [23]. IL-4 was expressed in the striatum of control rats, but not increased after ICH. IL-4 and IL-13 bind to IL-4Rα and IL-13Rα1 chains in the type II receptor [83], and while it has been reported that IL-4 and IL-13 mRNA and protein levels in the brain are highly variable [86], IL-4 receptors are found on microglia and astrocytes [87]. IL-4Rα and IL-13Rα1 were up-regulated after ICH, but after a delay in the aged animals.

Consistent with the expression of stimuli and receptors that evoke alternative macrophage/microglia activation in vitro, ICH increased the expression of several markers: ARG1, MRC1/CD206, CD163, CCL22, CHI3L1, MMP12. ARG1 is notable because it competes with iNOS for l-arginine, but while iNOS generates potentially harmful levels of nitric oxide, ARG1 produces l-ornithine, indirectly reducing nitric oxide. Further ornithine metabolism produces l-proline, which is a substrate for collagen synthesis [54], and has been extensively studied in wound healing and pulmonary fibrosis. The massive, diffuse accumulation of collagen in the ICH lesion by day 7 suggests the presence of ARG1 activity and alternative activation. In aged animals, increases in ARG1, MRC1, and CD163 were delayed to between 3 and 7 days. Early induction of iNOS was also greatly reduced, and it is possible that ARG1 elevation was not needed to shift l-arginine metabolism away from nitric oxide production. Although delayed, the increase in MRC1 reached a much higher level on day 7. MRC1 roles in the brain are not known, but it is important for pinocytosis and endocytosis by peripheral macrophages [84, 88]; its expression is low during the acute, pro-inflammatory phase and later up-regulated to help remove serum high-mannose glycoproteins [89]. Future studies will be needed to address whether increased MRC1 in aged rats corresponds with, and regulates, white matter-infiltrating macrophages and microglia.

MMP12 is induced in IL-4 stimulated macrophages [90, 91] and is a recently proposed marker of alternative activation [92]. MMP12 was the most highly up-regulated of the genes we analyzed after ICH. In young rats, there were marked increases in MMP12 expression [21, 27, 93] (present study), and at early times (1 and 3 days), it was present in astrocytes and around fragmented brain microvessels [21]. Here, we show that in aged rats, the increase was delayed (7 days), which correlates with the delayed expression of the alternative activation-related genes, MRC1, CD163, IL-4Rα, and IL-13Rα. While MMP12 functions in the brain are not known, there is some evidence for a deleterious role after ICH. MMP12-deficient mice had less microglia/macrophage recruitment to the hematoma and better sensorimotor recovery [93], and minocycline decreased MMP12 expression [21, 27], reduced edema and BBB leakiness [21], and improved the functional outcome [27]. Future studies will be needed to address whether age-related changes in MMP12 reflect changes in alternatively activated macrophages/microglia.

Microglia/macrophage activation is thought to contribute to the worse injury and poorer outcomes in aged animals after ICH [10, 73]. There are numerous reports that treatments inhibiting microglia/macrophage activation decrease hematoma volume and edema formation while improving neurological outcome [71, 94, 95]. However, the present study further illustrates the complexity of the innate inflammatory response in the first week after experimental ICH in which the induction of pro-inflammatory, potentially harmful mediators coincides temporally with many resolving and beneficial molecules. While this first study is necessarily descriptive, many time- and age-dependent changes were seen in the expression of inflammation-related genes, with many being reduced or delayed in aged rats. This evidence of a shift in the balance of inflammatory processes paves the way for further studies, including determining when, where, and in which cell types they are produced and how this expression changes with aging.

## Electronic Supplementary Material

Below is the link to the electronic supplementary material.

ESM 1 (PDF 263 kb)
