# Supplementary material for: Age-Related Comparisons of Evolution of the Inflammatory Response After Intracerebral Hemorrhage in Rats
Source: Transl Stroke Res. 2012 Mar 16;3(Suppl 1):132–46. doi: 10.1007/s12975-012-0151-3 (PMC3372776; doi:10.1007/s12975-012-0151-3)
Supplement: Supplementary file 1 — (PDF 263 kb) [file 12975_2012_151_MOESM1_ESM.pdf]

Title: Age-related comparisons of evolution of the inflammatory response after intracerebral hemorrhage in rats

Journal: Translational Stroke Research

Authors: Starlee Lively[1] and Lyanne C. Schlichter[1, 2]

Affiliations: 1. Toronto Western Research Institute, University Health Network

2. Department of Physiology, University of Toronto

Address for correspondence: Prof. L. Schlichter

E-mail: schlicht@uhnres.utoronto.ca

Legend for Online Resource 1

Summary of cellular events during evolving inflammation and tissue damage after intracerebral hemorrhage (ICH) induced by stereotaxic injection of bacterial type IV collagenase into the striatum of young adult rats. The findings are based on our previously published studies (see manuscript for discussion and references). The box locations are meant to illustrate the lesion edge versus core; i.e., the images are not taken from those exact positions. Scale bars: 100 μm

Fluorescent labels: DAPI (4'-6-diamidino-2-phenylindole was used to stain cell nuclei. Mouse monoclonal antibodies against: a neuronal nuclear marker (NeuN), glial fibrillary acidic protein (GFAP) to identify astrocytes, myelin basic protein (MBP) to label white matter bundles. Rabbit polyclonal antibodies against: myeloperoxidase (MPO) to label neutrophils, ionized calcium-binding adapter-1 (Iba1) to label microglia and macrophages, degraded MBP (dMBP) to identify damaged white matter tracts, collagen type IV (CgIV) which is a major constituent of the basal lamina of cerebral blood vessels. Extravasation of immunoglobulin G (IgG) was detected with a FITC-conjugated monoclonal anti-IgG antibody. Secondary antibodies were: DyLightTM488-conjugated AffiniPure donkey anti-mouse IgG, DyLightTM594-conjugated AffiniPure donkey anti-rabbit or anti-mouse

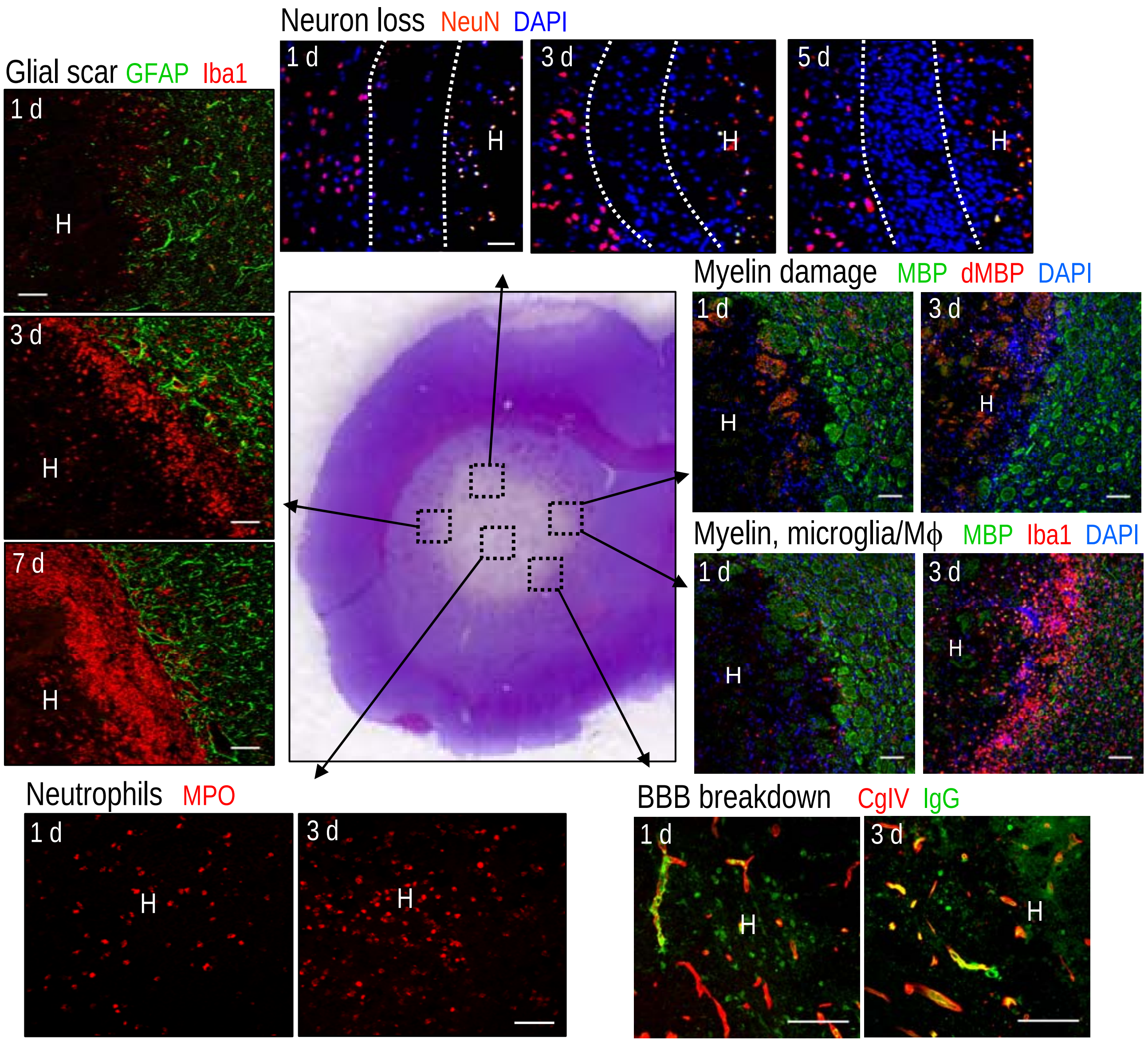

Lively et al.
Online resource 1
